# Chronic Lung Diseases and the Risk of Depressive Symptoms Based on the China Health and Retirement Longitudinal Study: A Prospective Cohort Study

**DOI:** 10.3389/fpsyg.2021.585597

**Published:** 2021-07-22

**Authors:** Xueling Ren, Shengshu Wang, Yan He, Junsong Lian, Qian Lu, Yanhong Gao, Yuling Wang

**Affiliations:** ^1^Department of Respiratory, The Second Medical Center of Chinese PLA General Hospital, Beijing, China; ^2^Institute of Geriatrics, Beijing Key Laboratory of Aging and Geriatrics, National Clinical Research Center for Geriatrics Diseases, The Second Medical Center of Chinese PLA General Hospital, Beijing, China; ^3^Department of Occupational Disease Treatment, Medical Center of The Second Artillery, Beijing, China; ^4^The Second Medical Center of Chinese PLA General Hospital, Beijing, China; ^5^Department of Nursing, Chinese PLA General Hospital, Beijing, China

**Keywords:** chronic lung diseases, depressive symptoms, cohort study, association, risk variants

## Abstract

Chronic lung diseases (CLDs) can reduce patients’ quality of life. However, evidence for the relationship between CLD and occurrence with depressive symptoms remains unclear. This study aims to determine the associations between CLD and depressive symptoms incidence, using the data from the China Health and Retirement Longitudinal Study (CHARLS). CLD was identified via survey questionnaire and hospitalization. The follow-up survey was conducted in 2018 and depressive symptoms were assessed by the 10-item Center for Epidemiological Studies Depression Scale (CES-D-10). A total of 10,508 participants were studied with an average follow-up period of 3 years. A total of 2706 patients (25.8%) with newly diagnosed depressive symptoms were identified. The standardized incidence rate of depressive symptoms in baseline population with and without chronic pulmonary disease was 11.9/100 and 8.3/100 person-years, respectively. The Cox proportional risk model showed that CLD was a significant predictor of depressive symptoms (HR: 1.449, 95% CI: 1.235–1.700) after adjusting for covariates, and the HRs of depressive symptoms were higher in those participants with current smoking (HR: 1.761, 95% CI: 1.319–2.352), men (HR: 1.529, 95% CI: 1.236–1.892), living in rural areas (HR: 1.671, 95% CI: 1.229–2.272), with dyslipidemia (HR: 1.896, 95% CI: 1.180–3.045), and suffering from comorbidity (HR: 1.518, 95% CI: 1.104–2.087) at baseline survey. CLD was an independent risk factor of depressive symptoms in China. The mental health of CLD patients deserves more attention.

## Introduction

Depression has rapidly become a major public health problem worldwide. There are more than 8 million deaths and approximate 350 million people suffering from depressive symptoms to different degree every year (Whiteford et al., 2010; Walker et al., 2015; Vigo et al., 2016; World Health Organization, 2017). Depressive disorders can cause a series of adverse consequences, such as disability, cognitive decline, functional damage, and even suicide (Steffens et al., 2006; Whiteford et al., 2013; Ismail et al., 2017). Depressive disorders contribute to the major global disease burden, and its contribution is rising (Whiteford et al., 2013). The years lived with disability (YLDs) caused by depressive disorders has increased by 37.6% from 1990 to 2010. Moreover, the global burden of mental disease may be seriously underestimated (Vigo et al., 2016).

In China, the disease burden of depressive disorders has been increasing gradually, and it becomes a huge and growing burden (Yang et al., 2013; Baxter et al., 2016). From 1990 to 2010, major depression was ranked the second leading cause of disability for both sexes and all ages in China (Yang et al., 2013; Zhou et al., 2019). Additionally, unlike the developed countries, the general cognition awareness of mental illness is still at a low level in China (Xu et al., 2017; Huang et al., 2019).

The risk factors of depression over the 40 years of research were cognitive ability and cognitive process; unbearable stressors; certain sociodemographic factors, such as being female and living in a rural area; heredity; and so on (Seedat et al., 2009; Stein et al., 2014; Hammen, 2018). In addition, recent researches have presented that chronic lung disease (CLD) was the independent risk factor for depressive symptoms (Xiao et al., 2018; Sampaio et al., 2019). However, no conclusive evidences were well-established; the majority of studies were based on cross-sectional or convenience samples studies (van den Bemt et al., 2009; Crump et al., 2014). No related evidence based on prospective, national sample studies was found in China. Hence, a representative large-sample cohort study of Chinese middle-aged and elderly people from the community was used to determine the relationship between baseline CLD status and depressive symptoms incidence and also to explore the influence of sociodemographic factors and comorbidity on this association.

## Materials and Methods

### Subjects

The current data in this study originated from the China Health and Retirement Longitudinal Study (CHARLS) in 2015 wave 3 (Chen et al., 2019). This database is based on a randomized multistage stratified probability proportional sampling investigation, covering 150 counties and 450 communities/villages in 28 provinces and cities in China, involving more than 17,000 subjects aged 45 or above from 10,000 families. Details of the cohort design, methods, and follow-up plan for CHARLS have been published previously (Zhao et al., 2014). All relevant information of demographics, previous history of disease, and the Center for Epidemiological Studies Depression Scale (CES-D-10) were collected by trained staff. The follow-up survey was completed in 2018, and incident depressive symptoms were defined as the outcome event. CHARLS was approved by the Biomedical Ethics Committee of Peking University and written informed consent was signed by each respondent.

**FIGURE 1:**
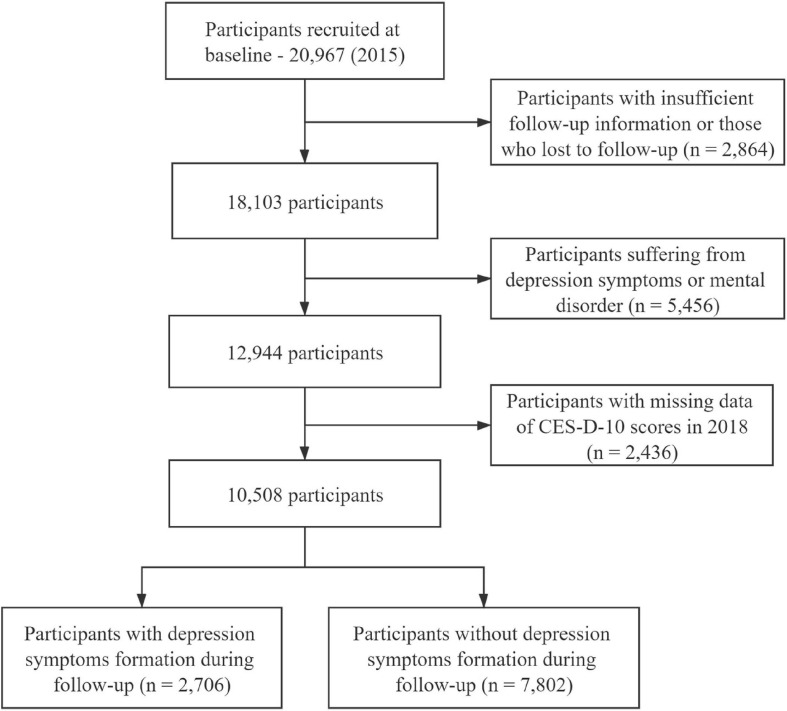
Study flow chart using the data from CHARLS (2015–2018).

In the baseline survey wave, a total of 20,967 participants were included. After excluding those who lacked baseline information, were lost to follow-up, had been diagnosed with depressive symptoms at baseline survey, and were missing important variable data, a total of 10,508 eligible participants fully qualified for data analysis were enrolled. Figure 1 shows the flow chart of research participants.

### Definitions

The CES-D-10 scale is administered to evaluate the depression symptom severity (Li et al., 2019). This scale consists of three items analyzing symptoms of depressed affect, five items assessing somatic symptoms, and two items estimating symptoms of positive affect. Response options for each item ranged from 0 to 5 (“rarely or none of the time” to “all of the time”), and the scores of items 5 and 8, which estimate positive affect, are reversed. The total score ranges from 0 to 30, where the higher the score, the more serious the depressive symptoms. Depressive symptoms were assessed using CES-D-10 at baseline and 3 years follow-up, and CES-D-10 with a cumulative score of 10 or more was considered to be of depressive symptoms in this study (Luo et al., 2018; Li et al., 2019).

Diagnoses of CLD, cardiovascular disease (CVD), chronic kidney disease (CKD), dyslipidemia, diabetes, and digestive system disease were recorded based on the self-report. Smoking status was dichotomized into “yes” or “no,” and alcohol drinking status was divided into three categories (drink more than once a month, drink but less than once a month, and none) according to the questionnaire. To better understand the age differences between the CLD and occurrence of depressive symptoms, age was categorized into <60- and ≥60-year groups. Body mass index (BMI) was calculated as weight (kg)/height (m)^2^. Blood pressure was measured twice in the right arm and the average SBP and DBP values were taken from the current study. According to the Chinese household registration system “hukou,” the residential type of the participants was classified into two groups: urban and rural. Participant educational level was categorized into three groups according to years of school (illiterate, 0 years; primary school, 1–6 years; middle school and above, ≥10 years). Presence of comorbidity was defined as no comorbidity versus comorbidity without or with CLD.

### Statistical Analyses

Statistical interpretation of data was performed using packages R (The R Foundation)^1^ and EmpowerStats (X&Y Solutions, Inc., Boston, MA, United States)^2^. The continuous data were expressed as mean and standard deviation (SD), and the differences between participants with and without CLD were evaluated by Student’s *t* test. Categorical data were expressed as number of cases and percentage, and the differences of whether the participants suffered from CLD were analyzed by Chi-square test. Univariate Cox proportional hazards model was used to evaluate the associations between baseline variable and depressive symptoms. Multivariate adjusted Cox proportional hazards model was performed to estimate HRs (95% CI) of CLD for depressive symptoms incidence. In multivariate model 1, adjusted variables were age, educational level, BMI, residential type, and marital status, and the adjusted variables of model 2 were model 1 plus smoking, drinking, digestive disease, CVD, and CKD. Stratified analysis was performed by gender, age group, rural/urban, and smoking status using multivariate Cox proportional hazards model. Interactions between baseline variables and whether participants suffered from CLD were tested by adding each multiplicative factor in the Cox proportional hazards model. *P* values less than 0.05 were considered statistically significant.

## Results

Baseline prevalence characteristics of CLD of 10,508 participants are shown in Table 1. The average age was 57.62 ± 9.44 years, 66.9% of the participants lived in rural areas, 85.2% had middle education, 33.8% were currently smoking, and 30.1% were drinking more than once a month. Regarding whether they are suffering from CLD, participants with CLD had older age, lower body mass index (BMI), lower systolic blood pressure (SBP), and lower smoking and drinking rates (*P* < 0.05). The prevalence of CLD was higher in men than in women, and higher in rural than in urban areas (*P* < 0.05). The participants with CLD had higher prevalence of CVD, CKD, and digestive disease than those without CLD (*P* < 0.001).

**TABLE 1:** Baseline characteristics of participants (*N* = 10,508).

Characteristic	Total	Chronic lung diseases		*P* value
		No (*n* = 9731)	Yes (*n* = 777)	
**Mean ± SD**				
Age, (years)	57.62 ± 9.44	57.23 ± 9.38	62.49 ± 8.87	<0.001
BMI (kg/m^2^)	24.19 ± 3.26	24.23 ± 3.23	23.73 ± 3.55	<0.001
SBP (mmHg)	128.62 ± 17.19	128.49 ± 17.10	130.14 ± 18.27	0.010
DBP (mmHg)	76.75 ± 9.52	76.74 ± 9.47	76.84 ± 10.15	0.782
***N* (%)**				
**Gender (*n*, %)**				<0.001
Male	5489 (52.24%)	5010 (51.48%)	479 (61.65%)	
Female	5019 (47.76%)	4721 (48.52%)	298 (38.35%)	
**Residential type (*n*, %)**				0.005
Rural	7026 (66.86%)	6471 (66.50%)	555 (71.43%)	
Urban	3482 (33.14%)	3260 (33.50%)	222 (28.57%)	
**Educational level (*n*, %)**				0.044
Illiteracy	872 (8.30%)	791 (8.13%)	81 (10.42%)	
Primary school	686 (6.53%)	629 (6.46%)	57 (7.34%)	
Middle school and above	8950 (85.17%)	8311 (85.41%)	639 (82.24%)	
**Marital status (*n*, %)**				<0.001
Married	9602 (91.55%)	8922 (91.88%)	680 (87.52%)	
Widowed/divorced/single	886 (8.45%)	789 (8.12%)	97 (12.48%)	
**Dyslipidemia (*n*, %)**				<0.001
Yes	852 (8.11%)	762 (7.83%)	90 (11.58%)	
No	9656 (91.89%)	8969 (92.17%)	687 (88.42%)	
**Diabetes (*n*, %)**				<0.001
Yes	454 (4.32%)	402 (4.13%)	52 (6.69%)	
No	10054 (95.68%)	9329 (95.87%)	725 (93.31%)	
**Tumor/cancer (*n*, %)**				0.0012
Yes	73 (0.69%)	62 (0.64%)	11 (1.42%)	
No	10435 (99.31%)	9669 (99.36%)	766 (98.58%)	
**CVD (*n*, %)**				<0.001
Yes	1012 (9.63%)	838 (8.61%)	174 (22.39%)	
No	9496 (90.37%)	8893 (91.39%)	603 (77.61%)	
**CKD (*n*, %)**				<0.001
Yes	474 (4.51%)	399 (4.10%)	75 (9.65%)	
No	10034 (95.49%)	9332 (95.90%)	702 (90.35%)	
**Digestive disease (*n*, %)**				<0.001
Yes	1970 (18.75%)	1698 (17.45%)	272 (35.01%)	
No	8538 (81.25%)	8033 (82.55%)	505 (64.99%)	
**Smoking (*n*, %)**				<0.001
Current	3124 (33.77%)	2891 (34.11%)	233 (29.99%)	
Former	1371 (14.82%)	1186 (13.99%)	185 (23.81%)	
Non-smoker	4757 (51.42%)	4398 (51.89%)	359 (46.20%)	
**Drinking (*n*, %)**				0.498
Drink more than once a month	3146 (30.12%)	2919 (30.19%)	227 (29.21%)	
Drink but less than once a month	967 (9.26%)	902 (9.33%)	65 (8.37%)	
No	6333 (60.63%)	5848 (60.48%)	485 (62.42%)	
**Comorbidity**				<0.001
No	9522 (90.62%)	9188 (94.42%)	334 (42.99%)	
Comorbidity without CLD	551 (5.24%)	543 (5.58%)	8 (1.03%)	
Comorbidity with CLD	435 (4.14%)	0 (0.00%)	435 (55.98%)	

*SBP, systolic blood pressure; DBP, diastolic blood pressure; CVD, cardiovascular disease; CKD, chronic kidney disease.*

During the 31,254 person-years of follow-up, a total of 2706 participants were diagnosed with depressive symptoms. The incidence in 3 years was 25.8% (95% CI: 25.0–26.6%), and the incidence density (per 100 person-years) was 8.6% (95% CI: 8.3–8.9%). The standardized incidence rate of depressive symptoms in baseline population with and without CLD was 11.9% (95% CI: 10.6–13.3%) and 8.3% (95% CI: 8.0–8.6%), respectively. The incidence density of depressive symptoms was significantly higher in the participants with CLD than those without CLD (*P* < 0.05) (Table 2).

**TABLE 2:** Incidence of depressive symptoms according to baseline chronic lung diseases status.

Variables	Baseline chronic lung diseases status		Total (*n* = 10,508)
	Yes (*n* = 777)	No (*n* = 9731)	
**Depression**			
Number of incident cases	277	2429	2706
Incidence (%)	35.6 (32.3–39.1)	30.0 (29.1–30.9)	25.8 (25.0–26.6)
Total person-years	2331	29193	31524
Incidence rate (per 100 person-years)	11.9 (10.6–13.3)	8.3 (8.0–8.6)	8.6 (8.3–8.9)

**FIGURE 2:**
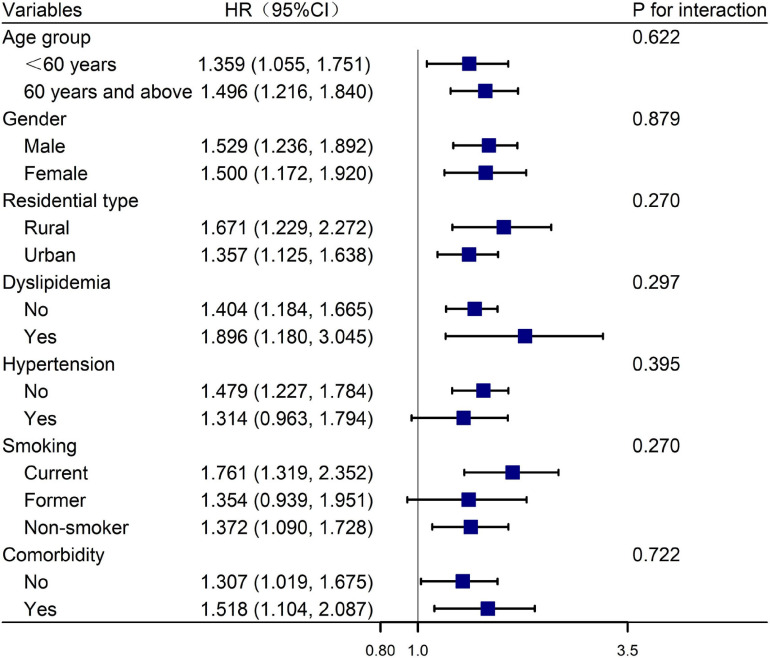
Forest plot of the relationship between baseline CLD status and depressive symptoms incidence. The blue square represents the HR value, and the line across the blue square represents the 95% confidence interval.

Table 3 shows the univariate analysis for depressive symptoms incidence. Table 4 presents the HRs of baseline CLD for depressive symptoms incidence during the follow-up and the subgroup analysis of the incidence of depressive symptoms according to smoking status, gender, age group, residential type, hypertension, dyslipidemia, and baseline comorbidity. After adjusting for age, gender, educational level, marital status, BMI, residential type, smoking, drinking, and baseline prevalence of CVD, CKD, and digestive disease, multivariate Cox regression showed that the adjusted HRs of depressive symptoms incidence caused by CLD was 1.449 (95% CI: 1.235–1.700). In the subgroup analysis, after the adjustment, compared with the general population, the adjusted HRs of depressive symptoms incidence were higher in those who were currently smoking, male, more than 60 years, living in rural areas, suffering from hypertension and dyslipidemia, and patients with comorbid conditions at baseline; the adjusted HRs were 1.761 (95% CI: 1.319–2.352), 1.529 (95% CI: 1.236–1.892), 1.496 (95% CI: 1.216–1.840), 1.671 (95% CI: 1.229–2.272), 1.479 (95% CI: 1.227–1.784), 1.896 (95% CI: 1.180–3.045), and 1.518 (95% CI: 1.104–2.087), respectively. There was no interaction between the subgroups (Figure 2).

**TABLE 3:** Univariate analysis for depression.

Covariate	Statistics	HR (95% CI)	*P* value
Age, (years)	57.62 ± 9.44	1.006 (1.002, 1.011)	0.007
BMI (kg/m^2^)	23.81 ± 3.89	0.982 (0.967, 0.997)	0.023
SBP (mmHg)	128.62 ± 17.19	0.999 (0.996, 1.001)	0.279
DBP (mmHg)	76.75 ± 9.52	0.995 (0.991, 1.000)	0.039
**Gender**			<0.001
Male	5489 (52.24%)	Ref.	
Female	5019 (47.76%)	1.599 (1.464, 1.746)	
**Marital status**			<0.001
Married	9602 (91.55%)	Ref.	
Widowed/divorced/single	886 (8.45%)	1.427 (1.231, 1.655)	
**Residential type**			<0.001
Urban	3482 (33.14%)	Ref.	
Rural	7026 (66.86%)	1.512 (1.372, 1.666)	
**Educational level**			
Illiteracy	872 (8.30%)	Ref.	
Primary school	686 (6.53%)	0.730 (0.590, 0.903)	0.004
Middle school and above	8950 (85.17%)	0.533 (0.461, 0.617)	<0.001
**CLD**			
No	9731 (92.61%)	Ref.	
Yes	777 (7.39%)	1.665 (1.428, 1.942)	<0.001
**CVD**			
No	9496 (90.37%)	Ref.	
Yes	1012 (9.63%)	1.443 (1.255, 1.659)	<0.001
**CKD**			
No	10034 (95.49%)	Ref.	
Yes	474 (4.51%)	1.559 (1.284, 1.894)	<0.001
**Digestive disease**			
No	8538 (81.25%)	Ref.	
Yes	1970 (18.75%)	1.646 (1.481, 1.829)	<0.001
**Smoking**			
Current	3146 (30.12%)	Ref.	
Former	967 (9.26%)	0.899 (0.772, 1.047)	0.169
Non-smoker	6333 (60.63%)	1.307 (1.178, 1.450)	<0.001
**Drinking**			
Drink more than once a month	3146 (30.12%)	Ref.	
Drink but less than once a month	967 (9.26%)	0.987 (0.830, 1.174)	0.884
None	6333 (60.63%)	1.362 (1.232, 1.506)	<0.001
**Comorbidity with CLD**			
No	9522 (90.62%)	Ref.	
Yes	986 (9.38%)	1.884 (1.643, 2.161)	<0.001
**Comorbidity**			
No	9522 (90.62%)	Ref.	
Comorbidity without CLD	551 (5.24%)	1.716 (1.433, 2.055)	<0.001
Comorbidity with CLD	435 (4.14%)	2.115 (1.737, 2.576)	<0.001

**TABLE 4:** HRs and 95% CI of baseline CLD status for depressive symptoms incidence.

Variable	Crude model		Model 1		Model 2	
	HR (95% CI)	*P* value	HR (95% CI)	*P* value	HR (95% CI)	*P* value
Total	1.665 (1.428, 1.942)	<0.001	1.613 (1.379, 1.887)	<0.001	1.449 (1.235, 1.700)	<0.001
Currently smoking	2.042 (1.544, 2.701)	<0.001	1.945 (1.464, 2.583)	<0.001	1.761 (1.319, 2.352)	0.001
Male	1.745 (1.421, 2.142)	<0.001	1.681 (1.364, 2.072)	<0.001	1.529 (1.236, 1.892)	<0.001
≥60 years	1.706 (1.396, 2.084)	<0.001	1.682 (1.374, 2.060)	<0.001	1.496 (1.216, 1.840)	0.001
Rural	1.892 (1.409, 2.540)	<0.001	1.857 (1.375, 2.507)	<0.001	1.671 (1.229, 2.272)	0.001
Non-hypertension	1.706 (1.425, 2.043)	<0.001	1.643 (1.367, 1.974)	<0.001	1.479 (1.227, 1.784)	<0.001
Dyslipidemia	2.094 (1.331, 3.295)	0.001	2.038 (1.281, 3.241)	0.003	1.896 (1.180, 3.045)	0.008
Comorbidity	1.297 (1.002, 1.680)	0.048	1.251 (0.960, 1.630)	0.093	1.518 (1.104, 2.087)	0.010

*Model 1: adjusted for age, educational level, BMI, residential type, and marital status. Model 2: adjusted for age, educational level, BMI, residential type, marital status, smoking, drinking, digestive disease, CVD, and CKD.*

## Discussion

This nationally representative, community-based CHARLS (2015–2018) found that baseline CLD status was independently associated with depressive symptoms incidence, and this causality was more pronounced in those participants who were currently smoking, male, more than 60 years, living in rural areas, and suffering from hypertension and dyslipidemia and comorbid conditions at baseline.

Depressive symptoms are complex and a common mental order with high incidence in the general population, and people with persistent depressive symptoms have a higher likelihood of developing depression or even schizophrenia (McCann et al., 2018). Studies on the risk factors of depressive symptoms have been a hot topic. Due to the decline of life quality caused by CLD leading to psychological burden, the negative effects of CLD on psychological influence are increasingly focused, but with large ranges in different populations (Matte et al., 2016). CLDs are complex, common, and multifactorial chronic diseases including chronic obstructive pulmonary disease (COPD), asthma, pneumonia, and so on. Data from WHO Study on global Ageing and adult health (SAGE) Wave 1 (2007–2010) showed that participants with baseline CLD exhibited a 3.74-fold higher incidence of developing depressive symptoms (Lotfaliany et al., 2018). A multicenter prospective cohort study in Korea found that CLD represents an independent risk factor for depression (Lee et al., 2013). The follow-up results of CHARLS presented in our study were in agreement with most of the previous studies. Middle-aged and elderly people with baseline CLD status was independently associated with depressive symptoms incidence, providing evidence from Chinese large-sample population with national representation for this association.

This study has shown that the CLD participants living in rural areas have higher incidence of depressive symptoms than those living in urban areas. In China, the prevalence of depression shows marked regional differences, which in urban and rural areas are 16% and 30%, respectively (Yang et al., 2019). This may be related to the poor economic development and medical and health services in rural areas (Qiu et al., 2020). This effect is more pronounced among CLD participants who are living in rural areas. No significant gender difference existed in the association between CLD and depressive disorders.

Evidence has shown that harmful gasses and particulates produced by smoking can lead to airflow restriction, which was the main cause of depressive symptoms induced by CLD (Taghizadeh, 2018). Despite the inconsistent direction between smoking and depression, their close relationships are unquestionable. This suggests that smoking may increase the association between CLD and depressive symptoms incidence. The results of this study showed that the incidence was higher in those who are suffering from CLD and were more than 60 years old. Older adults are more prone to suffer from worse chronic diseases, higher prevalence of physical disability, poorer cognitive activity, and lower socioeconomic status, which might cause more severe depressive symptoms. Comorbidity imposed a heavy disease burden on patients and families, predicting higher likelihood of depression incidence (Essau, 2011; Boden and Foulds, 2016).

The present study has several strengths. CHARLS is a national representative, reliable, large-sample prospective cohort study of participants aged more than 45 years old. In addition, this study provides the prospective evidence about the influence of baseline CLD status on depressive disorders during the follow-up.

Still, some limitations exist. Firstly, all disease information was self-reported, and there also existed the possibility of under-reporting, which weakens the effect. Secondly, rate of lost to follow-up in the current study and depressive symptoms at baseline were relatively high, and the follow-up duration was also relatively short, which might reduce the magnitude of association between CLD and depressive symptoms, to a certain extent. Thirdly, the information about treatment and control of CLD and depressive symptoms was lacking. The incidence of depressive symptoms between participants with and without treatment cannot be compared.

## Conclusion

The results of this study showed that CLD was an independent risk factor for depressive symptoms in the middle-aged and elderly people, and the causality was more pronounced among those who were currently smoking, male, more than 60 years, living in rural areas, and suffering from hypertension and dyslipidemia and comorbid conditions. This suggested that the mental health of CLD patients deserves special attention.

## Data Availability Statement

The datasets presented in this study can be found in online repositories. The names of the repository/repositories and accession number(s) can be found below: http://opendata.pku.edu.cn/.

## Ethics Statement

The studies involving human participants were reviewed and approved by the Peking University. The patients/participants provided their written informed consent to participate in this study.

## Author Contributions

XR, SW, YG, and YW were involved in the conception and design of the work. XR and SW contributed to writing the manuscript. XR, SW, YH, JL, and QL contributed to data arrangement and statistical analysis. All authors contributed to the article and approved the submitted version.

## Conflict of Interest

The authors declare that the research was conducted in the absence of any commercial or financial relationships that could be construed as a potential conflict of interest.

## Footnotes

1 http://www.R-project.org

2 http://www.empowerstats.com
